# End-of-life expenditure on health care for the older population: a scoping review

**DOI:** 10.1186/s13561-024-00493-8

**Published:** 2024-03-01

**Authors:** Ewa Kocot, Azzurra Ferrero, Shibu Shrestha, Katarzyna Dubas-Jakóbczyk

**Affiliations:** 1 Health Economics and Social Security Department, Institute of Public Health, Faculty of Health Sciences, Jagiellonian University Medical College, Krakow, Poland; 2 Ospedale Michele e Pietro Ferrero, Verduno-Azienda Sanitaria Locale CN2, Alba-Bra, Italy; 3 Kathmandu, Nepal

**Keywords:** End-of life expenditure, Older people, Proximity to death, Decedents, Survivors

## Abstract

**Background:**

The existing evidence shows that the pattern of health expenditure differs considerably between people at the end-of-life and people in other periods of their lives. The awareness of these differences, combined with a detailed analysis of future mortality rates is one of the key pieces of information needed for health spending prognoses. The general objective of this review was to identify and map the existing empirical evidence on end-of-life expenditure related to health care for the older population.

**Methods:**

To achieve the objective of the study a systematic scoping review was performed. There were 61 studies included in the analysis. The project has been registered through the Open Science Framework.

**Results:**

The included studies cover different kinds of expenditure in terms of payers, providers and types of services, although most of them include analyses of hospital spending and nearly 60% of analyses were conducted for insurance expenditure. The studies provide very different results, which are difficult to compare. However, all of the studies analyzing expenditure by survivorship status indicate that expenditure on decedents is higher than on survivors. Many studies indicate a strong relationship between health expenditure and proximity to death and indicate that proximity to death is a more important determinant of health expenditure than age per se. Drawing conclusions on the relationship between end-of-life expenditure and socio-economic status would be possible only by placing the analysis in a broader context, including the rules of a health system’s organization and financing. This review showed that a lot of studies are focused on limited types of care, settings, and payers, showing only a partial picture of health and social care systems in the context of end-of-life expenditure for the older population.

**Conclusion:**

The results of studies on end-of-life expenditure for the older population conducted so far are largely inconsistent. The review showed a great variety of problems appearing in the area of end-of-life expenditure analysis, related to methodology, data availability, and the comparability of results. Further research is needed to improve the methods of analyses, as well as to develop some analysis standards to enhance research quality and comparability.

**Supplementary Information:**

The online version contains supplementary material available at 10.1186/s13561-024-00493-8.

## Background

People close to death constitute a small part of populations, but health care expenditure on them is usually disproportionately high and this is especially the case for the population of older people. For example, Gastaldi-Menager et al. assessed that in France, decedents represent annually 3.5% of the group aged 65, but they account for 10.3% of total health expenditure on this group [1]; Medicare spending on decedents in the US was estimated to be 21%, 22%, or even 27.4% of total yearly spending ([2–4] respectively). Naturally, the highest number of deaths is observed among the older population: in Europe in 2019 over 75% of deaths concerned people aged 65 and over [5].

Population ageing is a universal phenomenon around the world. According to the UN World Population Prospect, the share of the population aged 65 and over in the world will increase by about 6.5% points (to 15.9%) in the next 30 years to rover 1.5 billion people [6]. The growth of the older population and its share of the total will be observed in all countries, without any exception. Taking into account the growing number of older people, health expenditure analyses in this population group are becoming more and more important for adequate health policy decisions.

The existing evidence shows that the pattern of health expenditure differs considerably between people at the end of life (EoL) and people in other periods of their lives. Expenditure usually increases rapidly in the time close to death; consequently decedents’ expenditure is much higher than survivors’ (e.g. [7, 8]). The awareness of these differences, combined with detailed analysis of future mortality rates is one of the key pieces of information needed for health spending prognoses. Not taking into account in prognostic analyses the different patterns of spending in the last and not-last periods of life may lead to overestimation of results (e.g. [8–10]). As most deaths are observed in older age, EoL expenditure analyses for this group are especially important.

Although the older population is often referred to in general, in many aspects, this group is not homogeneous. There are differences in health status and socio-economic characteristics which may influence health care needs and accessibility. Subsequently, this variation translates into differences in the health expenditure level. Understanding the relationship between health expenditure and different variables is important, e.g., for planning actions to reduce health inequalities [11]. The factors influencing health expenditure, and the strength of their association with expenditure may differ for decedents and survivors. For example, an important factor, cause of death, appears in decedents analysis; additionally, the structure of care is changing and nursing homes, hospices, and palliative care are becoming more important [12, 13]. Therefore, expenditure in the last period of life should be analyzed separately.

The general objective of this review was to identify and map the existing empirical evidence on the end-of-life expenditure on health care for the older population. Specifically, the review aimed to identify the relevant empirical studies on the analyzed topic, to provide an overview of their characteristics and results, and consequently, to help build and systemize the knowledge around the topic. To the authors’ best knowledge, no extended review related to this topic and focused on the older population, has so far been conducted.

## Methods

A systematic scoping review was performed. The methodological framework of the review was based on the studies of Arksey & O’Malley and Levac et al. [14, 15]. The PRISMA-ScR (Preferred Reporting Items for Systematic Reviews and Meta-Analyses extension for Scoping Reviews) checklist was followed in the reporting of the review [16] (Additional file 1). The project has been registered through the Open Science Framework [17].

### Research questions

To achieve the objective of the study, the following specific research questions were defined:

1. What kind of health care settings/types of care are the subject of research on EoL expenditure for the older population?
2. What definitions of the period of EoL are adopted in analyses?
3. What are the differences between expenditure on decedents and survivors in the older population?
4. What are the differences of EoL expenditure by age and other characteristics of older people?
5. What limitations are connected with research on this topic?

### Identifying relevant literature

The following databases were searched for eligible studies: (1) MEDLINE (Ovid), (2) EMBASE, (3) the Web of Science Core Collection, (4) Scopus, and (5) ProQuest. The searches were conducted in June 2022. No limits concerning publication dates were set. Three topic groups of search terms were defined, connected by the Boolean operator “AND”: (1) population, (2) timing, and (2) costs. The example search string used in the MEDLINE-Ovid database is presented in Table 1 and the full search strategy is reported in Additional File 2. To identify additional literature to include, reference lists of relevant articles and reviews were also searched.

**Table 1:** Search terms (MEDLINE-OVID)

Topic group	Search string
Population	aged[MeSH Terms] OR aged OR elderly[MeSH Terms] OR elder* OR older* OR senior* OR geriatrics[MeSH Terms] OR geriatric* OR pensioner* OR 65 OR 70 OR 75 OR 80 OR 85 OR 90 OR “old age” OR (old adj1 (population or person* or people or patient*)) OR (retired adj (population or people or person*)) OR “dependent population”
Timing	“end of life” OR “death related” OR “related to death” OR “last adj4 life” OR “end stage of life” OR “terminal year” OR “final adj4 life” OR “mortality related” OR “related to mortality”
Costs	cost* OR expenditure* OR spending* OR expense* OR financing OR “financial resources”

### Study selection

**Fig. 1:**
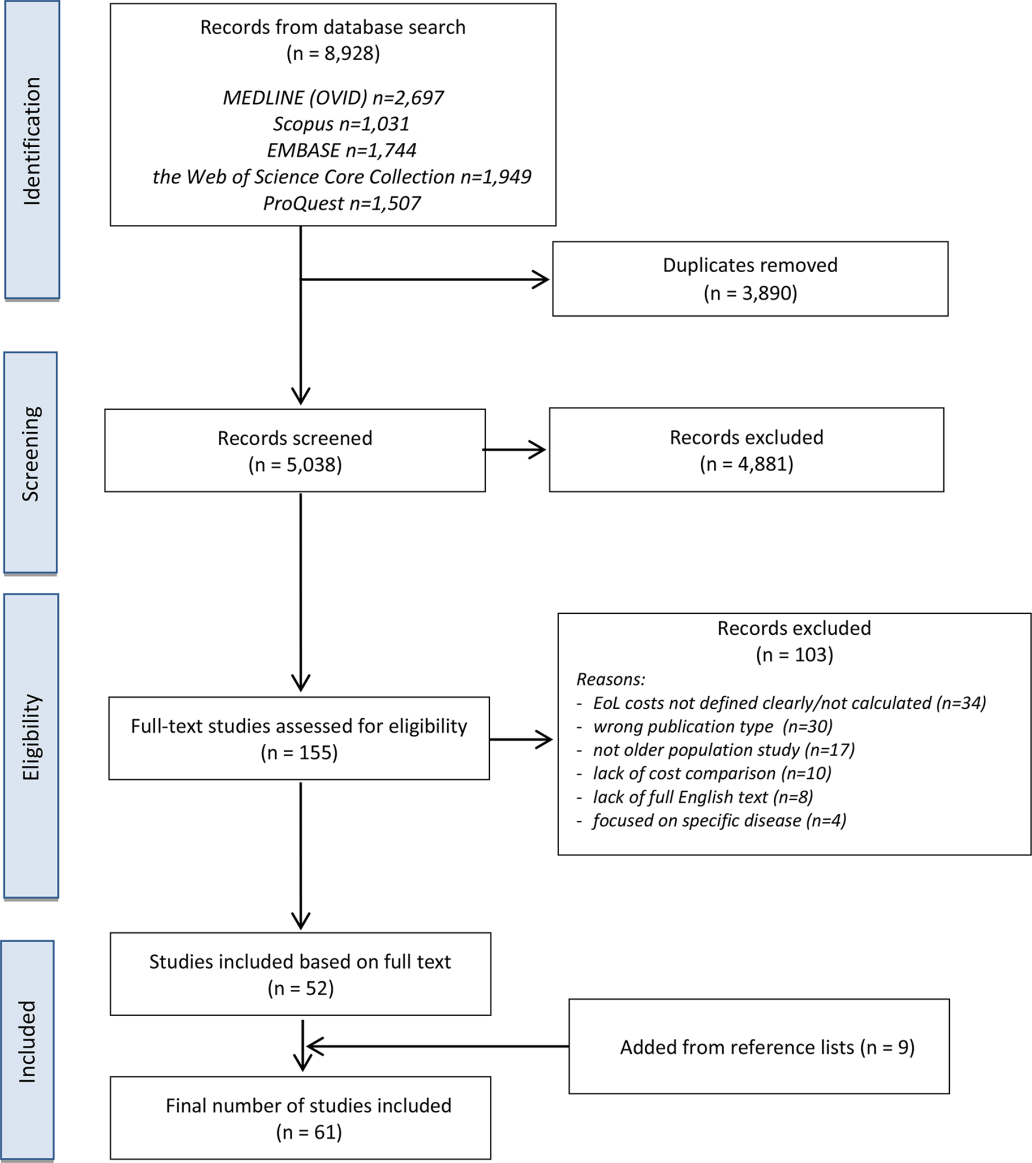
PRISMA flow diagram

The first step of study selection was screening the titles and abstracts of all earlier-identified articles to eliminate studies that do not address the objective of the review or its research questions. Initially, two researchers (authors) screened a randomly chosen sample of items (10% of all) and discussed the results. The procedure was repeated until the researchers achieved a 90% agreement of decisions in a given sample. After that, the remaining studies were screened by one researcher. The full texts of potentially relevant articles were screened based on inclusion-exclusion criteria by the two authors independently to select the final items to include. In the case of conflicting eligibility decisions, the third author was asked to give an additional opinion and the final decision was made on the basis of a majority. Reasons for exclusion are stated in the PRISMA flowchart (Fig. 1). The web application Ryyan was used to organize the study selection process.

The inclusion criteria:

1. results for older people available (analysis regards older people only or results for the older population are clearly separated; “older people” is understood broadly: 60+/65+/70+/etc.);
2. results available (a) for sub-groups of the older population, distinguished by age or other characteristics or (b) for non-EoL group to compare or (c) for younger population to compare;
3. EoL strictly defined;
4. Analysis related to health care expenditure;
5. EoL expenditure calculated (quantitative research);
6. Peer-reviewed original research article or review;
7. The full text available in English.

The exclusion criteria:

1. Analysis of palliative/terminal care in general, without a strict definition of EoL;
2. Results presented for the older population, but without division into subgroups and without calculation for non-EoL group/younger population to compare;
3. Case-study analysis;
4. Analysis conducted for treatment of a specific illness;
5. Conference abstract, note, communication, opinion;
6. Full text in language other than English.

### Data extraction

In the process of data extraction, a similar procedure as in the case of screening the titles and abstracts was used: the data extraction for a random 10% study sample was done by two researchers independently and discussed. The procedure was repeated until the researchers achieved a 90% consistency in the obtained data. After that, data for the remaining studies were extracted by one researcher.

The following data were extracted:

1. General study characteristics (author/s, year of publication, journal, study year/s, country of research);
2. Cohort characteristics;
3. Objective of the study;
4. EoL definition;
5. Methods and outcome measures (e.g., study design, type of statistical analysis used, indicators used);
6. Data type (administrative, survey);
7. Cost types/setting (e.g., hospital, primary care, medicine costs);
8. Limitations stated by the authors;
9. Key findings and conclusions.

### Collating, summarizing, and reporting the results

The included studies were categorized according to the characteristics of the expenditure they analyzed, taking into account the type of care/setting (e.g., inpatient care, outpatient care, nursing homes, hospices) and a payer (insurance, government, individual). The studies were also classified based on their adopted EoL period and methods of analysis. The main results presented by the authors in the studies have been reported in several categories, depending on what kind of associations are analyzed (the relationships between older decedents expenditure and age, proximity to death, socio-economic variables, health status, type of care) or with what the older decedents expenditure is compared (e.g., survivors expenditure).

## Results

### Search results

The databases search identified 8,928 relevant items. After removing duplicates, 5,038 of them were screened by title and abstract. After the title and abstract screening, 155 papers qualified for a full text analysis. At this stage, 103 studies were excluded for not meeting the inclusion criteria, and finally 52 papers were included. The reasons for exclusion were lack of clear definition or calculation of EoL costs (*n* = 34), publication type not meeting the criteria (*n* = 30), non-older population study (*n* = 17), lack of cost comparison (*n* = 10), lack of full text in English (*n* = 8) and study focused on a specific disease (*n* = 4). The references of all included papers were screened, which resulted in adding another nine papers to the final list. Finally, 61 papers were included for analysis. The search process is presented in the PRISMA flow diagram (Fig. 1).

### General characteristics of the included studies

More than half of the included studies were published in the last ten years, in the period of 2013 - June 2022 (*n* = 33; 54.1%). Only seven publications are dated before the year 2000. The years with the highest number of studies are 2015, 2016, and 2020 (*n* = 5).

The studies were conducted in 22 different countries, with more than a third of the studies (*n* = 22; 36.1%) including research results from the United States (US). Adding five studies from Canada, North America represents 44.3% of the research. 37.7% of the studies were conducted in Europe, but only one is from Central and Eastern Europe (Hungary). The rest of the studies come from Asia (*n* = 8) and Oceania (*n* = 5), with only one from Africa. Two papers present results of multinational studies, conducted in three countries ( [18, 19]: the US, the United Kingdom, and Ireland).

Table 2 presents the list of included studies by year and country, with full reference data.

**Table 2:** List of included studies with year/s and country of study

Reference number	First author/s and publication year	Date of study (including sample data time)	Country of study
[20]	Brameld et al. 1998	1985–1994	Australia
[21]	Kardamanidis 2007	2002–2003	Australia
[22]	Gielen et al. 2010	July 2005–June 2006	Belgium
[23]	Demers 1998	1991	Canada
[24]	Guerin et al. 2019	1. April 2020–31. March 2013	Canada
[25]	Hollander 2009	2003–2004	Canada
[26]	McGrail et al. 2000	1987–1988, 1994–1995	Canada
[27]	Tanuseputro et al. 2015	April 2010 - March 2013	Canada
[28]	Zhu et al. 2018	January-December 2015	China
[29]	Hansen et al. 2020	2013–2017	Denmark
[30]	Häkkinen et al. 2008	1997–2002	Finland
[31]	Bell-Aldeghi et al. 2022	2017	France
[1]	Gastaldi-Menager et al. 2016	2008–2013	France
[32]	Koczor-Keul 2017	2014	Hungary
[33]	Alipour et al. 2022	March 2013 and March 2014	Iran
[34]	Moore et al. 2017	2006–2009, 2009–2012	Ireland
[35]	Shmueli et al. 2010	2004	Israel
[36]	Hashimoto et al. 2010	2000–2004	Japan
[37]	Teraoka et al. 2021	April 2010 - March 2015	Japan
[38]	Boo et al. 2020	2009–2013	Korea
[39]	Hyun et al. 2016	2010–2014	Korea
[40]	van Vliet & Lamers 1998	1988–1994	Netherlands
[10]	Blakely et al. 2014	2007–2011	New Zealand
[41]	Blakely et al. 2015	2007/08 to 2009/10	New Zealand
[42]	Scott et al. 2021	2016–2018	New Zealand
[43]	Melberg et al. 2013	2010	Norway
[44]	Ranchod et al. 2015	2008–2013	South Africa
[45]	Hanratty et al. 2007	2002	Sweden
[46]	Felder 2001	1981–1992	Switzerland
[47]	Felder et al. 2000	1987–1992	Switzerland
[48]	Outreville 2001	1995–1997	Switzerland
[49]	Panczak et al. 2017	January 2008-December 2010	Switzerland
[50]	Wyl et al. 2018	2008–2010	Switzerland
[51]	Zweifel et al. 1999	1983–1992	Switzerland
[52]	Liu et al. 2002	1999	Taiwan
[53]	Geue et al. 2015	1972–2007	UK
[54]	Hazra et al. 2018	2010–2014	UK
[55]	Jayatunga et al. 2019	2011–2017	UK
[56]	Luta et al. 2020	2010–2017	UK
[18]	Higginson et al. 2020	not clear	UK, Ireland, USA
[19]	Yi et al. 2020	not specified	UK, Ireland, USA
[57]	Bird et al. 2002	1993–1998	USA
[58]	Davis et al. 2016	2011–2012	USA
[2]	Duncan et al. 2019	2015–2016	USA
[59]	Gozalo et al. 2015	2004 and 2009	USA
[60]	Hanchate et al. 2009	2001	USA
[4]	Hogan et al. 2001	1993–1998	USA
[61]	Holland et al. 2014	2007–2011	USA
[3]	Hoover et al. 2002	1992–1996	USA
[62]	Kelley 2016	2000–2010	USA
[63]	Khandelwal et al. 2019	2002–2014	USA
[64]	Lubitz & Prihoda 1984	1976–1978	USA
[65]	Lubitz & Riley 1993	1976, 1980, 1985, 1988	USA
[66]	McGarry & Schoeni 2005	1993	USA
[67]	Riley & Lubitz 2010	1978–2007 (1998–2000 excluded)	USA
[68]	Scitovsky 1988	January 19,830 August 1984	USA
[69]	Shugarman et al. 2004	1993–1999	USA
[70]	Sullivan et al. 2017	2008	USA
[71]	Yang et al. 2003	1992–1998	USA
[72]	Zuckerman et al. 2016	2010	USA
[73]	Miller et al. 2004	July-December 1999	USA

Note: ordered by the countries alphabetically

### Objectives of the studies

In more than 80% of the included studies (*n* = 50; 82%) objectives were defined only in a very general way, as investigation/exploration/evaluation/measurement of EoL costs. In 26 of these articles authors declared that they aimed to investigate the relationship between EoL costs and factors other than proximity to death, age or gender (which are commonly taken into account). These other factors mentioned in the objective were for example health conditions, income, geographical location, language, ethnicity, entitlements to health care. Authors of three studies formulated the objective as testing “red herring” hypothesis. In only 30% of studies (*n* = 18) there was information provided in the objective that results fully or partly concerned older people. There were additional specific objectives, beyond the general objective of the EoL costs analysis, defined in 11 studies. These are namely: to examine a financial risk of patients close to death (*n* = 2); to compare the expenditure predictions with and without including EoL costs (*n* = 2); to propose methodological improvement (in the area of data access and quality, methods of EoL costs estimation) (*n* = 2); to examine implications of research results for health care organization (*n* = 5).

### Samples, data, and methods used in the studies

**Fig. 2:**
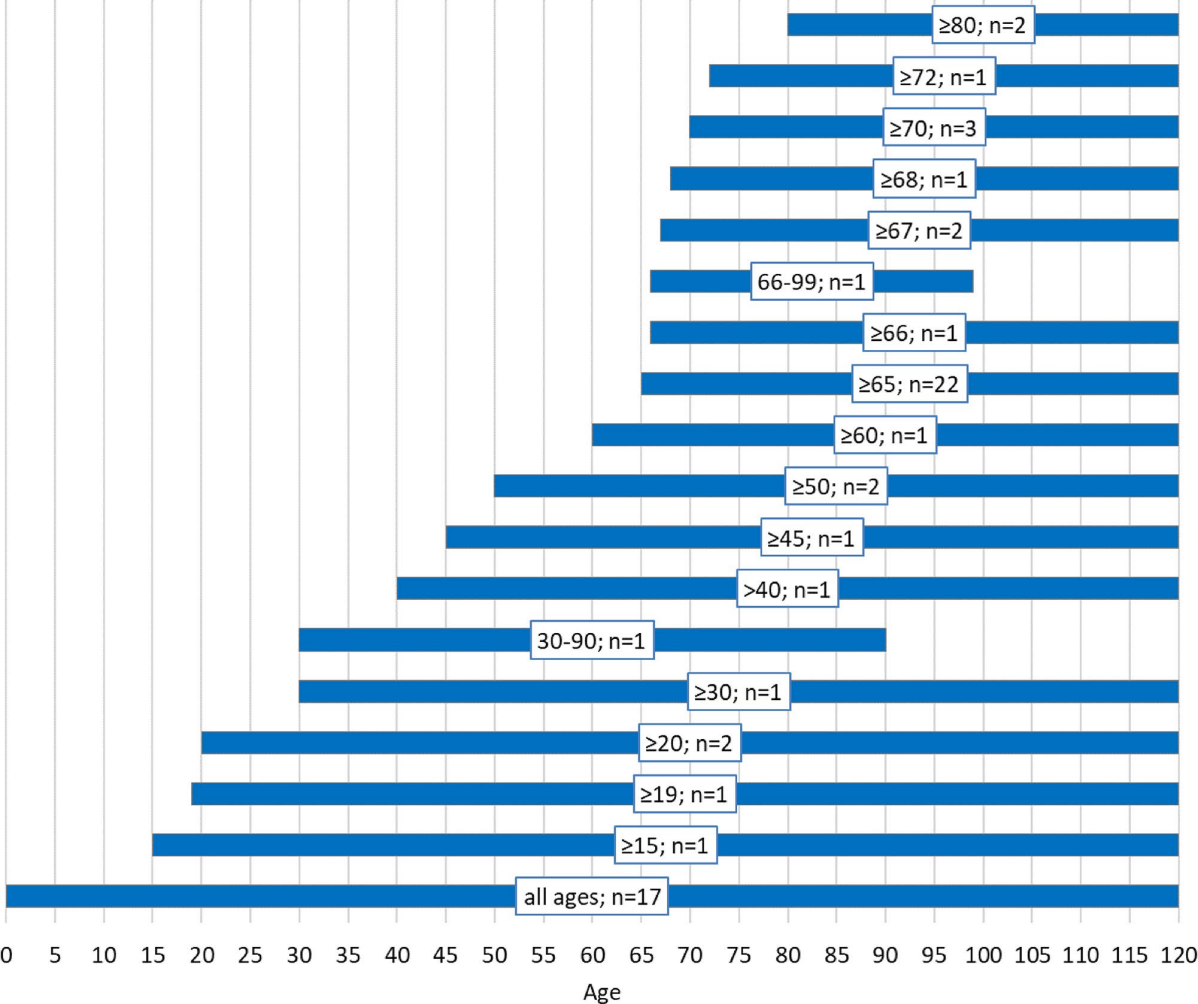
Number of studies with a given sample age. Note: in all cases where the study was not conducted exclusively on older population, the results are also presented for the older age group/groups separately, as this is the criterion of inclusion

According to the scope of this review, only studies regarding older people were included in the analysis. There are studies relating solely to the older population or studies relating to a wider age group in which results for older people are separately presented. Information about the age characteristics of the included studies samples is presented in Fig. 2.

More than half of the studies (*n* = 34; 55.7%) were conducted exclusively on older population groups, at the age of 60 or above. The most common age group among these older group studies is aged 65 and above (*n* = 22; 64.7%). Four publications do not provide exact information about the sample’s age, but as these are studies concerning Medicare beneficiary decedents, it was assumed that the group under consideration is aged 65 and over [2, 4, 70, 72]. In 17 studies there are no limits concerning age and the sample includes people of any age (including older age groups). Only for two of the samples was an upper age limit defined [33, 58], all other age brackets are opened on the right. As defined in the research topic, samples always include a group of decedents, but in the case of about a third of the studies, the sample also contains a group of survivors for comparison (*n* = 23; 37.7%).

The vast majority of authors used exclusively administrative data in their studies (*n* = 51; 83.6%). In four studies (6.6%), only data derived from surveys were used, and six studies (9.8%) were based on both types of data – administrative and surveys. The type of data used (administrative or survey) is not specifically related to the types of costs assessed in a given study, since, for example, the information about individual out-of-pocket (OOP) spending can also be obtained from administrative sources (Boo et al. used a public insurer reports to assess the OOP copayments of insured persons [38]), and data concerning publicly funded health services can be derived from surveys as well (Hakkinen et al. and Yi et al. used a mortality follow-back survey to identify all EoL expenditure, including the public expenditure [19, 30]). Among ten studies based totally or partly on surveys, seven draw on data from existing surveys, conducted external to the given study [3, 42, 53, 62, 63, 66, 71]; three studies are based on data gathered via surveys developed specifically by authors of the given study [18, 19, 68]. The survey data used in two studies [42, 53] does not relate directly to health service costs/utilization, but to the health status and behavior of participants.

Nearly 40% of the studies are cross-sectional analyses (*n* = 24) and about 16% are exclusively longitudinal (*n* = 10). In the case of the other 27 studies, it is not possible to unequivocally define the type of research – they include elements of longitudinal and cross-sectional analyses (e.g., in one study, cost trends are analyzed based on time period, and in another part of this study these costs are analyzed by age groups, race, sex, and income). In more than half of the studies (*n* = 32, 52.5%), authors used advanced statistical methods for the analysis (e.g., advanced econometric models, multivariable fractional polynomial models, probit, and generalized linear models). In 18% of the studies (*n* = 11), relatively simple statistical methods were used (e.g., t-test, Chi^2^ test, Spearman correlation, analysis of variance, simple regression). In nearly 30% of the studies (*n* = 18), statistical methods were not used, except simple median, mean and/or standard deviation calculation. Detailed information regarding the samples and methods of each included study can be found in the Additional file 3.

### Characteristics of the analyzed expenditures

As defined in the aim of this review, all included studies relate to health expenditure in the last period of individuals’ lives. However, this period is defined differently across the studies. Most often, in 70.5% of the studies (*n* = 43) authors used for analysis the period of one year before death. Other authors used: three months, six months, and two years (13.1% each), three years (11.5%), and one month (4.9%). In five studies a longer period was analyzed: seven years (in one study), five years (*n* = 3), and four years (*n* = 1). However, the defined, longer period before death can be additionally divided into shorter periods for analysis, for example one (or more) year(s) into months [3, 35, 38, 55, 71] or quarters [46–53, 29, 39, 51], a three-year period into years [69, 71]. The expenditure in these sub-periods may be analyzed separately for each of them (e.g., expenditure in the first, second, third, etc. month/quarter before death [51, 71]) or cumulatively (e.g., expenditure in the last month/quarter before death, in the last two, last three, last four, etc. months/quarters before death [3, 35]). Periods shorter than one month were also used for analyses, but in one study only [73]: 0–2 days, 3–7 days, 8–14 days, and 15–30 days prior to death.

The costs analyzed in the studies relate to various types of services and settings; however, hospital costs can be found in all but two analyses (the costs of all prescribed medicines outside hospital [34], informal care costs [18]). The authors of 22 studies declare that spending on all services is included (in three of them, authors acknowledge drug prescription cost exclusion [58, 65, 67], but usually it is not the total spending in the given country, but is limited to a given payer/payers: individual [31, 63, 66], Medicare (e.g. [4, 58, 60]**)**, one or more insurers (e.g. [22, 46, 47]), central or local government [25, 27]. Total (or nearly total) expenditure for all payers is included in three studies [62, 68, 71]. About three-quarters of the research (*n* = 46) includes an analysis of outpatient care and nearly half of the research (*n* = 29, 47.5%) includes analysis of primary care, but some errors are possible in the estimation of these numbers because the authors could qualify primary care as outpatient care, not declaring separately that primary care is included. Drug prescription costs were analyzed in 34 studies (55.7%). In almost 20% of the studies (*n* = 12), the authors declare that expenditure on nursing homes and/or hospices is included in the analysis.

Nearly 60% of the analyses are conducted for insurance expenditure (public or private, covering all insurance systems in a given country or only selected insurers) (*n* = 36, 59%), 38.8% of them (*n* = 14) are related to the US Medicare/Medicaid insurance program. In nine studies (14.8%), the authors focused on governmental spending. In the case of 13 studies, no clear information about payers or all payers is included, and the analyzed expenditure scope is defined rather by setting or type of costs: hospital costs [19–21, 28, 43, 53], palliative care costs [19], medication prescription costs [55], all types of costs (wide scope, some exclusions possible) [29, 30, 54, 56, 68, 71]. Only three studies regard solely OOP payments [31, 63, 66]. In six analyses, costs incurred by patients as co-payments or other contributions to insurance or governmental payment for services are taken into account [25, 26, 38, 50, 62, 73]. Patients’ payments are also included in the analyses of all costs, indicated above. One study analyzed informal care costs exclusively, using an indirect method of cost estimation based on shadow prices [18].

### Areas of comparison

**Fig. 3:**
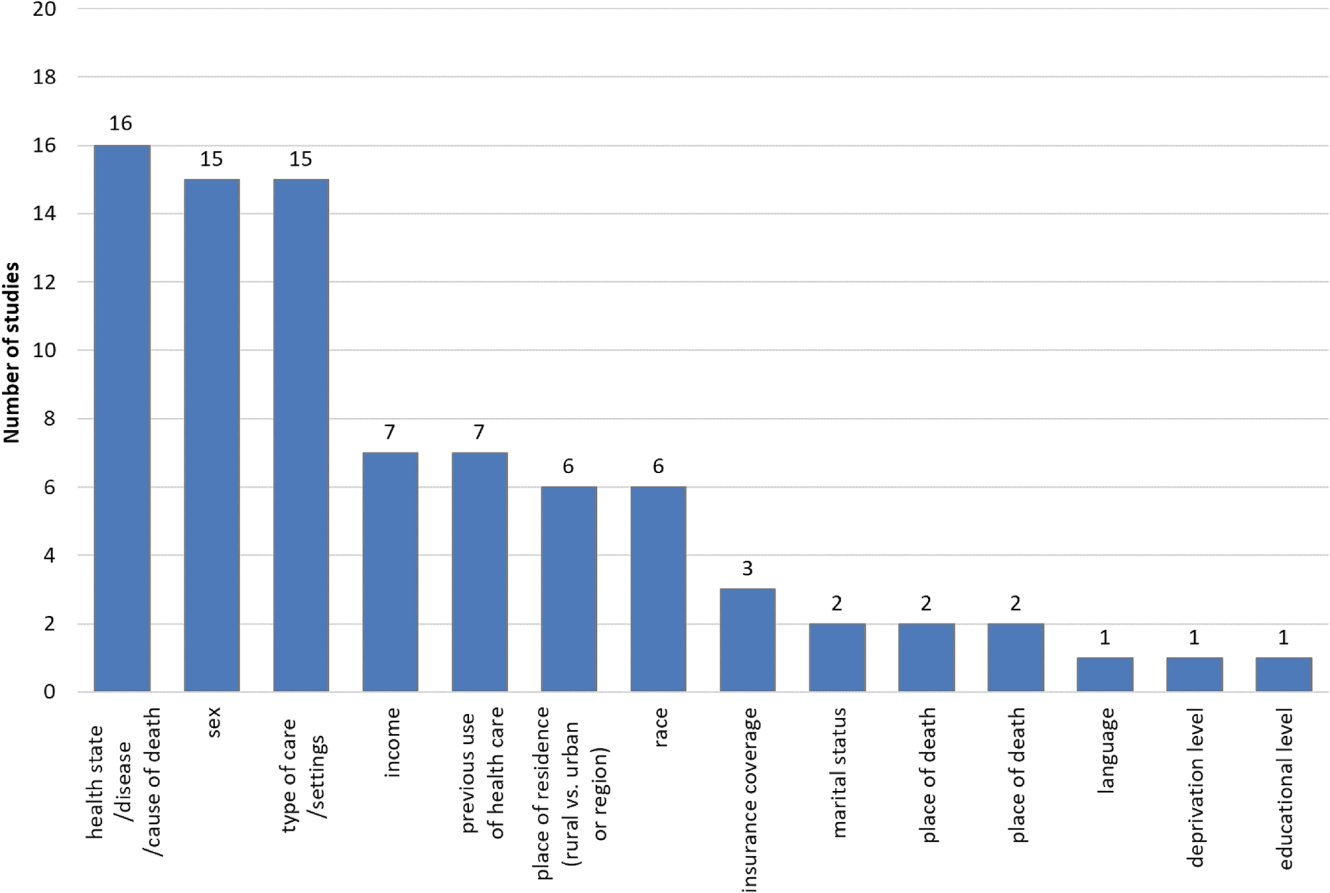
Bases of comparative analyses used in the included studies

To analyze health expenditure during the last period of life in the older population, the authors not only present its level, but also carry out diverse comparisons with expenditure among other groups of people (survivors or total population independently of survivorship), or based on various decedents characteristics. The most natural basis for comparison for decedents expenditure is analogous health expenditure on survivors; this analysis was conducted in over a third of the included studies (24 out of 61). The most common comparisons are related to age: the authors compare expenditure between age groups inside the older group of people and/or between the older and younger populations in nearly 80% of the studies (*n* = 48). In 26 studies, the analysis of expenditure in the last period of life depending on the proximity to death is provided, meaning the time prior to death is divided into smaller periods of time for comparison, e.g., a year into months or quarters. Other bases of comparative analyses taken into account in the included studies are presented in Fig. 3.

#### Expenditure on decedents vs. survivors

The results of the studies in terms of expenditure by survivorship status are very consistent and all of them (*n* = 19) indicate that expenditures on decedents are higher, or even much higher, than on survivors. Only in one analysis, for institutional care, were these expenditures assessed as nearly equal [36]. At the same time, lower spending on survivors than on decedents was observed for out- and inpatient care in the same study. Some studies provide information about the decedent to survivor ratio (meaning how many times expenditure on decedents is higher than on survivors). A comparison of this ratio between studies is not possible or very difficult as there are a lot of differences in the subject and scope of analysis, sample, and methods. For example, Hoover et al. [3] presented values from 2.43 to 6.29 (depending on the payer), and from 0.45 to 13.23 (depending on services). This ratio decreases with increasing age, as the gap between decedent and survivor expenditure is higher for younger people than for older people [26, 52, 54]. Looking at differences by the type of care, Riley & Lubitz [67] noticed that in the case of decedents compared to survivors, a higher share of total expenditure is spent on inpatient and skilled nursing care and lower for outpatient and physicians care.

#### End-of-life expenditure by age

In about 40% of the studies (*n* = 22), the results of the analysis show, in the scope of the expenditure covered by a given study, solely decreasing end-of-life expenditure trends as age increases in the older population (usually aged 65 and over). Additionally, the authors of two studies [22, 68] found a health spending decline in the oldest group (80+), and in one study [53] a decline in the group aged 70 and over (comparison between two age groups only). In two studies, the increase of expenditure in the last period of life was valid for all analyzed items [10, 33], and in 12 studies, mixed results are presented depending on different factors, including: (1) time to death (e.g., increasing with age for the 365 day period prior to death, but for 30, 90 and 180 days increasing until the age 74 and then decreasing: [25]), (2) type of care (e.g., decreasing with age for outpatient and inpatient care, stable for emergency care, increasing until age 90 and then decreasing for primary care: [55]), (3) use of care (e.g., for long-term care (LTC) users, stable expenditure for patients aged 74 and younger and increasing for older ones, declining spending on non-LTC users, spending increase for LTC users and non-LTC users as a one group: [30]), (4) payer (e.g. spending of Medicare decreasing with age and of non-Medicare increasing: [3]), (5) cause of death (e.g., decreasing expenditure trend for all causes of death except injuries: [21]) or (6) health state (e.g., for patients without comorbidities or impairment, spending was weakly associated with age, for other patients spending declined: [54]).

#### End-of-life expenditure by proximity to death

In most analyses on the relationship between end-of-life expenditure and proximity to death, the authors came to the conclusion that expenditure increased as people approached their death. Rapid growth can be observed especially in the period very close to death: the last quarter [2, 28, 39, 43, 70, 71], the last month [58, 21, 32, 43, 73], or even the last days [2]. In analyses of longer periods before death (two or more years) the higher level of expenditure is clearly visible in the last year of life compared to years farther from death [29, 30, 40, 69, 70]. Some authors presented different quantitative indicators showing this tendency. For example, Zhu et al. [28] estimated that hospital expenditure in the last quarter of a year of life accounts for 64% of hospital expenditure of the last year of life, and Melberg et al. [43] assessed the analogous share at 50%. Hyun et al. [39] stated that inpatient hospital expenditure is approximately 47% higher in the last quarter of life than in the 12th quarter before death, and Koczor-Keul et al. [32] estimated inpatient care expenditure to be nine times higher in the last month than in the 12th month prior to death. According to another study, total Medicare expenditure in the last month of life represented more than 40% of total expenditure of the last year [65].

However, another tendency was observed by Teraoka et al. [37]. Acoording to their study LTC expenditure increased only slightly in the period of 5 years before death and during the last four months it even decreased. A stable or nearly stable level of expenditure in the last period prior to death was also confirmed in the studies for home and institutional care [36], and pharmaceuticals [1]. Wyl et al. [50] classified expenditure in the group aged 66 and older (including outpatient care, inpatient hospital care, drugs, and nursing homes expenditure) into five trajectories, differing in the level and course of expenditure. Two of them (representing 51% and 18% of total end-of-life expenditure in this group) are characterized by a substantial decline of expenditure level in the last month of life. The results presented above suggest that there may exist areas and periods before death for which expenditures do not increase with the approach of death, but without assessing a quality of the conducted studies final conclusions must be drawn carefully.

#### Differences in end-of-life expenditure depending on demographics, socio-economic characteristics, and health status

In the analyzed studies there is no consistent picture regarding end-of-life expenditure by gender: in eight cases a clear difference in expenditure level between females and males was confirmed (although the results sometimes presented higher expenditure on males (*n* = 4), and sometimes on females (*n* = 4)), some authors presented mixed results of this analysis (*n* = 6), and no impact of gender on EoL expenditure level was indicated in one study. In six studies were racial and ethnic differences in end-of-life expenditure analyzed. Hanchate et al. stated [60] that Medicare expenditure in each of the last six months of life is higher for Black and Hispanic decedents than for Whites and this difference is largely due to higher use of intensive care services. Similar results for one and three years before death were presented by Hogan et al. and Shugarman et al. respectively [4, 69]. Contrarily, no ethnic differences were found by Scott et al. [42]. The results of analyses regarding the association between income/poverty and end-of-life expenditure are not unambiguous either. For example, in one study on Medicare decedents, higher expenditure for people living in areas with higher median household income was confirmed [69], but in another Medicare study higher expenditure in areas with higher poverty rate was shown [4].

Looking at analyses on the relationship between end-of-life spending and health status/cause of death, it is clearly visible that oncological diagnosis often causes higher expenditure in the last period of life than other conditions. This result was obtained for different types of care and funding sources [22, 29, 49, 56, 70]. The differences of a pattern of spending related to a cause of death were also observed. For cancer patients the decrease of health insurance expenditure during the last six months of life was observed after age 70, while for non-cancer patients only after age 90 [15]. Kardamanidis et al. [22] observed that injuries was the only cause of death for which costs in the last year of life did not decrease with age. People who died of injuries represented also the highest level of costs in the last month of life. Results of the study of Liu et al. [60] indicated that the average monthly expense increased rapidly in the last year of life for all causes of death, but in the case of cancer costs increased earlier than for a system failure and frailty, or chronic diseases. Two studies confirmed a positive association between expenditure and a number of conditions [56, 58], but another one showed that no relation exists [19].

#### End-of-life expenditure depending on type of care

Many researchers indicate that hospital care generates the highest share of end-of-life expenditure. For example, hospital costs account for 70–77% of total costs in the last year of life in the case of Medicare [64, 65] and for 56–60% when all payers in a given country are taken into account [29, 56]. Residential care spending is also a significant contributor to expenditure in the last period of life [2, 3, 29, 50]. Depending on the type of care, the pattern of EoL expenditure by age, proximity to death or other factors can be different (some examples are presented above).

### Limitations indicated by authors

In the majority of the studies (45 out of 61), the authors clearly indicated limitations. One of the most commonly reported limitations is connected with the scope of study in relation to: (1) payer: e.g., only insurance/government sources analyzed, only one plan of Medicare benefits taken into account, no OOP payment included (*n* = 14); (2) type of care/services/goods: e.g., only hospital/acute care costs taken into account, lack of LTC analysis, exclusion of medication prescription/primary care/mental health services (*n* = 13). Authors of 12 studies indicate factors not included in the analysis, which can affect study results: health status, cause of death, place of living and death, income, or gender. The next reported limitation, found in 12 studies, is related to samples used for analyses – due to a specific sample’s characteristics (e.g., connected to age, race, region, or entitlement to benefits), it may not represent the given country as a whole and may generate issues with the generalizability of the results. Another problem perceived by the authors is data quality and completeness (*n* = 11). In 11 studies, the authors indicate that in such analyses not only expenditure and use of services should be addressed, but also quality of care, its appropriateness, and accordance with patients’ preferences in the last period of life. The authors of 21 studies identify other weaknesses of the methods used in the studies, such as analyzing an insufficiently long period prior to death, a retrospective/observational character of a study (which can cause bias), lack of a control group, costs not fully related to terminal conditions, overly simple statistical methods used in a study, a data aggregation level that is too high, not fully adequate matching of analyzed decedent-survivor pairs. Information about main the limitations declared in each included study can be found in Additional file 3.

## Discussion

This review provides extensive information about the available evidence on EoL expenditure related to health care for the older population. There were 61 studies included in the analysis, coming from 22 countries and published between 1984 and June 2022. The vast majority of studies are based on administrative data; in only ten cases did authors use surveys to gather the data. In many studies it is difficult to clearly define their design (cross-sectional or longitudinal), as elements of both types are used. In more than half of studies, the authors used advanced statistical methods. Over 50% of the research considered only an older population sample, but in the case of studies conducted on a wider sample, and not only older age groups, solely the part of results for the older population is taken into account in this review.

The studies cover different kinds of expenditure in terms of payer, provider, and type of services, although most of them include analyses of hospital spending. This is not surprising, as hospital care accounts for the larger part of health expenditure, particularly in end-of-life care [7, 64, 65]. This may be caused by the fact that in many countries, insurance expenditure accounts for a significant part of the total, and/or data regarding this kind of spending is obtained from administrative sources, and therefore a long observation period for a large sample size is often available, data can be linked with different databases containing other useful information about patient, and gathering data does not generate additional costs [74–76]. As the main source of OOP data is surveys, it is more difficult to obtain and its quality can be lower [77]. Only in about a quarter of studies did the authors include partial or total individual patient expenditure in their analysis.

Although studies provide very different (often inconsistent) results, and additionally, due to the great variety of research scope, they are difficult to compare, all studies analyzing expenditure by survivorship status indicate that expenditure on decedents is higher, or even much higher, than on survivors. This is the only universal result indicated by the included studies, regardless of country, scope and methodology of research.

Drawing reliable conclusions on the relationship between EoL expenditure and socio-economic status would be possible after including only studies of confirmed quality, but additionally such kind of analysis would need to be placed in a broader context, including rules of a given health care system’s organization and financing. For example, it is not possible to draw conclusions on personal income differences in EoL expenditure without knowing what scope of care is guaranteed by public payers; a summary of the results on ethnic/racial differences is not possible without information about differences in the access to health care services.

The classification of included studies by the scope of analyzed expenditure is not easy. An expenditure item can be defined by the payer (e.g., insurance, government, individuals), by the service/good type which is financed (e.g., inpatient care service, drug prescription) or the setting where the service is provided (e.g., hospital, nursing home, primary care). As health care system organization and financing differ strongly between countries, data are reported in different ways, it is not always possible to create a uniform expenditure map for all studies. For example, in some studies, outpatient care provided in hospital can be included in hospital spending, but in others it is qualified as outpatient care, along with services provided outside hospitals. Also, long-term care can be understood very differently and covers different types of care provided in various settings, including or excluding hospital services or home care. Some authors used a setting as a base of analysis (e.g., hospital costs: [20, 28, 43, 53]), sometimes a payer is the starting point of expenditure identification (e.g., all spending of (a) given insurer’s/-s’ [22, 46, 47, 60] out-of-patient expenditure [31, 66] or total governmental spending [25, 27]. There are also studies with a more specific definition of analyzed items, connecting a payer and a setting aspect (e.g., all hospital expenditure borne by the insurer [32, 33, 39]). The analysis can also be based on the care type (e.g., all palliative care services, regardless of payer or setting [18, 19]). To facilitate review and comparison of results of end-of-life expenditure studies, it would be beneficial to use a unified system of health expenditure classification, that is, the National Health Accounts (NHA), which is the system used in almost all countries of the world. Authors’ information about analyzed expenditure, provided in the NHA’s nomenclature, would allow for much easier identification of its type.

Many authors of the included studies have addressed in their research a problem that has been discussed in the field of health economics for many years: does per capita health expenditure depend on calendar age or rather on the time remaining to death? Zweifel et al. over 20 years ago in their publication (which was, in fact, the starting point of the extensive discussion on this topic) stated that the econometric analysis of decedent expenditure did not confirm the effect of age on expenditure once the remaining lifetime is controlled for, and called the observed relationship between age and health care expenditure a “red herring” [51]. Hazra et al.’s [54] results showed that proximity to death is the strongest predictor of high costs and the association between costs and any other factors is much less significant when time to death is taken into account. The similar conclusion was made by other studies [26, 33, 34, 39, 56]. Geue et al. [53] found both time to death and age, but also the interaction between these two, to be significant predictors for expenditure in the last 12 quarters of life. Not taking into account in the expenditure prognosis the influence of time to death can lead to significant overestimation (e.g., [34, 78–81]). The awareness of the important impact of proximity to death on expenditure and, contrary to some research findings, the moderate impact of calendar age may be very important for health policy decisions. To rationalize future expenditure it aiming at controlling EoL treatment should be considered rather than focusing on population ageing [34].

However, although many studies indicate the leading role of proximity to death, the findings on the association between health care expenditure and the ageing process are still inconclusive, partly due to methodological issues of research [78]. One of the problems concerns the proper identification of expenditure to be analyzed. In all studies included in this review, the authors define a specific period before the death of an individual, and expenditure incurred during this period is considered end-of-life expenditure. Different lengths of this end-of-life period are adopted, from seven years to one month, but in all cases, this period is defined the same for the whole sample. However, the fact of death may impact the expenditure level for a longer or shorter period before death, depending mainly on a cause of death [78]. Moreover, some health services may be related to imminent death, and some not. While, for example, hospital costs incurred during a short period at the very end of life are highly likely to be related to the cause of death, by extending this time the risk of including expenditure not arising from the proximity of death increases. On the other hand, considering only a short period prior to death may result in spending actually related to death not being taken into account [43]. Some authors of the studies are aware of this issue: for example Panczak et al. [49] point out that costs from the last 12 months of life may include items unrelated to death, so they can only be considered a proxy for end-of-life costs. As the timing of death is, in most cases, unpredictable, analyzed spending (except in hospice) should not be understood to be generated by care delivered in anticipation of death, thus they are rather costs in the last year of life, not costs of dying or terminal illness [4, 44, 65, 67, 82]. Identifying expenditure directly related to death is a difficult task, especially if a wide scope of it is analyzed (not only hospice care or palliative care). Jayatunga et al. [55] tried to make such an assessment, matching decedents to survivors in terms of sex, age group, deprivation quintile, number of conditions, and residency, and then calculating costs differences in pairs. However, a vast majority of analyses do not even include any comments in this regard. To avoid a misinterpretation of results, information about what analyzed expenditures are strictly related to should be clearly indicated in such studies.

Another methodological problem that arises in the studies is related to the distinction between decedents and survivors. Some authors used a time of observation long enough to be sure that a given person’s status can be qualified as ‘survivor’ (e.g., [34, 36, 39, 55, 65, 67]. This means that preparing expenditure analysis for a given year, only a person who survived the whole next year could be considered a survivor. Otherwise, at least part of the expenditure incurred in the year under analysis should be qualified as decedent spending. However, in many studies this aspect is not taken into account (e.g., [1, 3, 4, 42–44, 48, 52, 64]), which may result in an underestimation of decedent expenditure and an overestimation of survivor expenditure. The underestimation of decedent expenditure may also be caused by using data about deaths and spending limited to a given calendar year, without any modeling or re-calculation (e.g., [1, 43]). People die in every month of a year, so for a person who died, for example, in March, expenditure incurred in the 9 last months of a previous year should be added.

This review is not free from limitations. Firstly, only publications in English were taken into account. Secondly, according to guidance on conducting a scoping review [15], a quality assessment of the included studies was not performed. This limited the possibility of synthesizing via more advanced methods the results of the included studies. However, due to the wide variety of methodological approaches, research scopes, and health care systems characteristics, such synthesis would be very difficult anyway.

This review showed that a lot of studies are focused on limited types of care, settings, and payers, showing only a partial picture of health and social care systems in the context of end-of-life expenditure for the older population. The analyses on social/community care and informal care are particularly lacking. It would be highly recommended to strengthen this area of research, especially as these types of care are very important in the last period of life. Obtaining comprehensive information about financial aspects of end-of-life health and social care in a country might significantly help in supporting adequate health policy decisions and providing people at the end of life with appropriate and efficient care. A very important aspect, on which the researchers should put a much stronger emphasis are patient’s preferences at the end of life. Some types of care, especially hospital care, may be burdensome and inappropriate for patients at the end of life, in comparison with alternatives which may be better and more consistent with patient preferences [7, 27, 83]. To properly support allocation decisions, policy-makers need to know whether high end-of-life expenditures ensure good quality and care appropriateness and are in line with patients’ preferences at the end of life. Research to date has not provided such information [22, 24, 36, 56, 60, 62, 67].

The results of this review shed light on some methodological challenges faced by researchers in the field of older people end-of-life expenditure analysis. Further efforts are needed to improve the identification and calculation of decedent/survivor expenditure, improve the quality and availability of data, and ensure greater international comparability of results. It would be worth checking what the main reason is for the complete or almost complete lack of research from South America, Central and Eastern Europe, and Africa.

## Conclusions

The results of studies on EoL expenditure for the older population conducted so far are largely inconsistent. The only consistent conclusion that emerges from all relevant studies is that EoL expenditure on older decedents is higher than expenditure on survivors. The results’ differences may be caused by the diversity of the scope of analyses as well as the diversity of the analyses’ contexts (e.g., health system design), so any comparisons should be made with caution.

The review showed a great variety of problems appearing in the area of EoL expenditure analysis, related to methodology, data availability, and the comparability of results. The results of the presented study constitute a good starting point for further research, to improve methods of analyses, as well as to develop some analysis standards in this field that can enhance research quality and comparability. It is also important that the results are interpreted and reported in a clear way, even if advanced statistical methods are used - the presentation of results should facilitate an application of results to health policy.

### Electronic supplementary material

Below is the link to the electronic supplementary material.

Additional file 1. Preferred Reporting Items for Systematic reviews and Meta-Analyses extension for Scoping Reviews (PRISMA-ScR) Checklist (table)

Additional file 2. Full search strategy (table)

Additional file 3. Overview of included studies (table)

## Data Availability

The data supporting the conclusions of this article is included within the article and its additional files.

## Abbreviations

EoL: end-of-life
LTC: long-term care
NHA: National Health Accounts
OOP: out-of-pocket
