# Supplementary material for: End-of-life expenditure on health care for the older population: a scoping review
Source: Health Econ Rev. 2024 Mar 1;14:17. doi: 10.1186/s13561-024-00493-8 (PMC10905877; doi:10.1186/s13561-024-00493-8)
Supplement: Supplementary file 1 — Additional file 1. Preferred Reporting Items for Systematic reviews and Meta-Analyses extension for Scoping Reviews (PRISMA-ScR) Checklist (table) [file 13561_2024_493_MOESM1_ESM.docx]

**Additional file 3**

**Overview of included studies^1)^**

| **Ref.** | **First author/s; publication year** | **Methods and sample** | | | | | **Expenditure characteristic** | | | **Main limitations stated by authors ^8)^** |
| --- | --- | --- | --- | --- | --- | --- | --- | --- | --- | --- |
|  |  | **Study design ^2)^** | **Statistical analysis level ^3)^** | **Data type ^4)^** | **Sample age** | **Survivors inclusion in sample (Y/N)** | **EoL period adopted ^5)^** | **Type of care/setting ^6)^** | **Payer ^7)^** |  |
| [56] | Moore et al. 2017 | CS, LT | 3 | A | 72+ | Y | 1Y, 2Y, 3Y | DP | N | F |
| [20] | Alipour et al. 2022 | LT | 3 | A | 30-90 | N | 2Y | H | I | S(T), F |
| [30] | Geue et al. 2015 | LT | 3 | A, S | 45+ | N | 3Y [Q] | H | N | S(T), D, M |
| [42] | Hyun et al. 2016 | LT | 3 | A | 20+ | Y | 3Y [Q] | H | I | N |
| [25] | Brameld et al. 1998 | CS, LT | 2 | A | 65+ | N | 1Y | H | N | S(T) |
| [44] | Kardamanidis 2007 | CS, LT | 1 | A | 65+ | N | 1Y, 1M, 3M, 6M | H | N | S(T) |
| [71] | Zhu et al. 2018 | CS, LT | 2 | A | total | N | 1Y | H | T | G |
| [54] | Melberg et al. 2013 | CS, LT | 1 | A | total | Y | 1Y | H | N | Q, M |
| [47] | Koczor-Keul 2017 | CS, LT | 2 | A | 65+ | N | 1Y | H | I | N |
| [62] | Scott et al. 2021 | CS | 3 | A, S | 80+ | Y | 1Y | H, NH | G | S(P), S(T), M |
| [55] | Miller et al. 2004 | CS, LT | 2 | A | 65+ | N | 1Y [M], several days periods | H, NH, Hp | G, OOP | G |
| [57] | Outreville 2001 | CS, LT | 1 | A | 30+ | Y | 2Y, 3M | H, Out | I | N |
| [48] | Liu et al. 2002 | CS | 1 | A | 20+ | Y | 1Y | H, Out | I | G |
| [27] | Demers 1998 | CS | 1 | A | 15+ | N | 1Y | H, Out, A | I | D |
| [1] | Gastaldi-Menager et al. 2016 | CS, LT | 1 | A | total | Y | 1Y, 3Y | H, Out, DP | I | N |
| [53] | McGrail et al. 2000 | CS, LT | 1 | A | 65+ | Y | 6M | H, Out, DP | G, OOP | M |
| [34] | Hakkinen et al. 2008 | CS, LT | 3 | A | 65+ | Y | 1Y, 2Y, 3Y, 4Y | H, Out, DP | T | N |
| [63] | Shmueli et al. 2010 | CS, LT | 3 | A | total | Y | 1Y [M] | H, Out, DP, A, E, D | I | F, M |
| [3] | Hoover et al. 2002 | CS, LT | 3 | A, S | 65+ | Y | 1Y [M] | H, Out, DP, HC, D, Hp, NH | I(M), I, OOP | D |
| [24] | Boo et al. 2020 | LT | 2 | A | 65+ | N | 1Y [M] | H, Out, DP, LTC | I, OOP | D |
| [67] | Teraoka et al. 2021 | LT | 3 | A | 70+ | N | 1Y, 5Y | H, Out, DP, LTC | I | G, D |
| [69] | Wyl et al. 2018 | LT | 3 | A | total | N | 1Y | H, Out, DP, NH | I, OOP | M |
| [68] | van Vliet & Lamers 1998 | CS, LT | 1 | A | total | Y | 7Y | H, Out, DP, R, D | I | N |
| [40] | Holland et al. 2014 | CS | 3 | A | 65+ | N | 1Y, 2.Y | H, Out, DP, SN, E | I | S(P), F, M |
| [2] | Duncan et al. 2019 | CS, LT | 1 | A | 65+ | N | 1Y, 3M, 6M | H, Out, HC, E, Hp, SN, MS | I(M) | N |
| [32] | Gozalo et al. 2015 | CS | 3 | A | 67+ | N | 1Y | H, Out, HC, Hp | I(M) | S(P), Q |
| [38] | Hashimoto et al. 2010 | CS, LT | 3 | A | 65+ | Y | 1Y | H, Out, LTC | I | G, Q, M |
| [58] | Panczak et al. 2017 | CS | 3 | A | 19+ | N | 1Y, 3M | H, Out, NH | I | F, D, Q, M |
| [36] | Hanratty et al. 2007 | CS | 3 | A | total | N | 1Y | H, Out, PC | G | N |
| [10] | Blakely et al. 2014 | CS | 1 | A | total | Y | 1Y, 6M | H, Out, PC, A, DP | G | S(P), S(T), D |
| [23] | Blakely et al. 2015 | CS | 1 | A | total | Y | 1Y, 6M | H, Out, PC, A, DP | G | S(P), S(T), D |
| [51] | Luta et al. 2020 | CS, LT | 3 | A | 60+ | N | 1Y, 1M | H, Out, PC, DP | T | F, G, Q, M |
| [43] | Jayatunga et al. 2019 | CS | 3 | A | 50+ | Y | 1Y [M] | H, Out, PC, E, SC | G | S(P), S(T), F, G, M |
| [22] | Bird et al. 2002 | CS | 2 | A | 65+ | N | 1Y | H, Out, SN, HC | I(M) | S(P) |
| [64] | Shugarman et al. 2004 | CS | 3 | A | 68+ | N | 3Y | H, Out, SN, HC, Hp | I(M) | F, M |
| [19] | Yi et al. 2020 | CS | 2 | S | 65+ | N | 3M | H, P, SC | T | S(T), G, M |
| [37] | Hansen et al. 2020 | CS, LT | 3 | A | 65+ | N | 5Y [Q] | H, PC, DP, HC, NH, SC | T | M |
| [39] | Hazra et al. 2018 | CS | 3 | A | 80+ | Y | 1Y | H, PC, E, DP, HC | T | S(T), F |
| [18] | Higginson et al. 2020 | CS | 3 | S | 65+ | N | 3M | informal care costs | N | D |
| [29] | Felder et al. 2000 | LT | 3 | A | total | N | 2Y [Q] | T | I | N |
| [73] | Zweifel et al. 1999 | LT | 3 | A | total | N | 2Y [M] | T | I | M |
| [35] | Hanchate et al. 2009 | CS, LT | 3 | A | 66+ | N | 6M | T | I(M) | S(P), D, Q |
| [41] | Hollander 2009 | CS, LT | 1 | A | total | N | 1Y, 1M, 3M, 6M | T | G, OOP | N |
| [65] | Sullivan et al. 2017 | CS, LT | 3 | A | 65+ | N | 1Y, 2Y | T | I(M) | F, M |
| [66] | Tanuseputro et al. 2015 | CS, LT | 2 | A | total | N | 1Y | T | G | S(P), S(T), G, Q |
| [70] | Yang et al. 2003 | CS, LT | 2 | A, S | 65+ | N | 3Y [M] | T | T | N |
| [49] | Lubitz & Prihoda 1984 | CS, LT | 1 | A | 67+ | Y | 1Y, 2.Y | T | I(M) | N |
| [59] | Ranchod et al. 2015 | CS, LT | 1 | A | total | Y | 1Y | T | I | N |
| [21] | Bell-Aldeghi et al. 2022 | CS | 3 | A | 65+ | N | 1Y, 3M | T | OOP | S(T), G |
| [31] | Gielen et al. 2010 | CS | 3 | A | >40 | N | 6M | T | I | S(P), F, Q |
| [33] | Guerin et al. 2019 | CS | 3 | A | total | N | 1Y | T | I | G, Q |
| [45] | Kelley 2016 | CS | 2 | A, S | 70+ | N | 5Y | T | I(M), I, OOP | S(P), Q, M |
| [46] | Khandelwal et al. 2019 | CS | 3 | S | 50+ | N | 1Y | T | OOP | D |
| [61] | Scitovsky 1988 | CS | 1 | A, S | total | N | 1Y | T | T | G, M |
| [72] | Zuckerman et al. 2016 | CS | 3 | A | 65+ | N | 6M | T | I(M) | M |
| [4] | Hogan et al. 2001 | CS | 2 | A | 65+ | Y | 1Y | T | I(M) | N |
| [52] | McGarry & Schoeni 2005 | CS | 1 | S | 70+ | Y | 2Y | T | OOP | N |
| [28] | Felder 2001 | LT | 3 | A | total | N | 1Y [Q] | T | I | N |
| [26] | Davis et al. 2016 | LT | 3 | A | 66-99 | N | 1Y | T (except DP) | I(M) | S(P), S(T), F |
| [50] | Lubitz & Riley 1993 | CS, LT | 1 | A | 65+ | Y | 1Y | T (except DP) | I(M) | S(P), M |
| [60] | Riley & Lubitz 2010 | CS, LT | 1 | A | 65+ | Y | 1Y | T (except DP) | I(M) | S(P), Q, M |

^1)^ Sorted by a Type of care/setting

^2)^ CS-cross-sectional, L-longitudinal

^3)^ Statistical analysis level: 1-lower level of statistical analysis (e.g. descriptive statistics, simple cost comparisons); 2-medium level of statistical analysis (e.g. t-test, analysis of variance, simple regression); 3-higher level of statistical analysis (e.g. advanced econometric models multivariable fractional polynomial models)

^4)^ A-administrative, S-survey

^5)^ the period before a death adopted in analysis: *n*Y-*n* years; *n*M-*n* months; 2.Y-second year; in the brackets information about additional dividing the main period into shorter ones: [M]-into months; [Q]-into quarters

^6)^ H-hospital; Out-outpatient; PC-primary care; DP-drug prescription; R-rehabilitation; NH-nursing homes; Hp-hospices; HC-home care; P-palliative care; A-ancillary services; E-emergency; LTC-long-term care; SN-skilled nursing facility; MS-medical supplies; D-dental services; SC-social care; T-all types of care

^7)^ I-insurance; I(M)-Medicare; G-governmental/public spending; OOP-out-of-pocket/individual; T-all payers; N-no payer defined

^8)^ S(P)-scope of study related to payer; S(T)-scope of study related to typ of care; F-factors not included; G-generalizability issues; D-data quality/completeness; Q-quality/appropriateness of care not included; M-other methodological issues; N-no limitations indicated
