# Supplementary material for: End-of-life expenditure on health care for the older population: a scoping review
Source: Health Econ Rev. 2024 Mar 1;14:17. doi: 10.1186/s13561-024-00493-8 (PMC10905877; doi:10.1186/s13561-024-00493-8)
Supplement: Supplementary file 3 — Additional file 3. Overview of included studies (table) [file 13561_2024_493_MOESM3_ESM.docx]

**Additional file 2**

**Full search strategy**

| **Database** | **Search string** |
| --- | --- |
| MEDLINE (Ovid) | (aged[MeSH Terms] OR aged OR elderly[MeSH Terms] OR elder* OR older* OR senior* OR geriatrics[MeSH Terms] OR geriatric* OR pensioner* OR 65 OR 70 OR 75 OR 80 OR 85 OR 90 OR “old age” OR (old adj1 (population or person* or people or patient*)) OR (retired adj (population or people or person*)) OR “dependent population”)AND(“end of life” OR “death related” OR “related to death” OR “last adj4 life” OR “end stage of life” OR “terminal year” OR “final adj4 life” OR “mortality related” OR “related to mortality”)AND(cost* OR expenditure* OR spending* OR expense* OR financing OR “financial resources”) |
| EMBASE | ('death related' or 'end of life' or 'related to death' or 'end stage of life' or 'terminal year*' or 'mortality related' or 'related to mortality' or ( last NEXT/4 life ) or ( final NEXT/4 life )):ab,ti AND (('cost*' or 'expenditure*' or ';spending*' or 'expense*' or 'financing' or 'financial resources'):ab,ti) AND ((aged or elder* or older* or senior* or geriatric* or pensioner* or 65 or 70 or 75 or 80 or 85 or 90 or 'old age*' or ( old NEXT/1 ( population or person* or people or patient* ) ) or ( retired NEXT/1 ( population or people or person* ) ) or 'dependent population'):ab,ti) |
| the Web of Science Core Collection | ((((((((TS=("death related")) OR TS=("end of life")) OR TS=("related to death")) OR TS=("end stage of life")) OR TS=(last NEAR/4 life)) OR TS=(final NEAR/4 life)) OR TS=("terminal year*")) OR TS=("mortality related")) OR TS=("related to mortality")AND(((((TS=(cost*)) OR TS=(expenditure*)) OR TS=(spending*)) OR TS=(expense*)) OR TS=(financing)) OR TS=(financial resources)AND(((((((((((((((TS=(aged)) OR TS=(elder*)) OR TS=(older*)) OR TS=(senior*)) OR TS=(geriatric*)) OR TS=(pensioner*)) OR TS=(65)) OR TS=(70)) OR TS=(75)) OR TS=(80)) OR TS=(85)) OR TS=(90)) OR TS=("old age")) OR TS=(old NEAR/1 (population or person* or people or patient*))) OR TS=(retired NEAR/1 (population or person* or people))) OR TS=("dependent population") |
| Scopus | (TITLE-ABS("end of life") or TITLE-ABS("death related") or TITLE-ABS("related to death") or TITLE-ABS(„terminal year*") or TITLE-ABS(last W/4 life) or TITLE-ABS(„end stage of life") or TITLE-ABS(final W/4 life) or TITLE-ABS(„mortality related") or TITLE-ABS(„related to mortality")) AND (TITLE-ABS(cost*) or TITLE-ABS(expenditure*) or TITLE-ABS(spending*) or TITLE-ABS(expense*) or TITLE-ABS(financing) or TITLE-ABS(financial resources)) AND  (TITLE-ABS(aged) or TITLE-ABS(elder*) or TITLE-ABS(older*) or TITLE-ABS(senior*) or TITLE-ABS(geriatric*) or TITLE-ABS(pensioner*) or TITLE-ABS(65) or TITLE-ABS(70) or TITLE-ABS(75) or TITLE-ABS(80) or TITLE-ABS(85) or TITLE-ABS(90) or (TITLE-ABS(old age*) or TITLE-ABS(old W/1 (population or person* or people or patient*)) or TITLE-ABS(retired W/1 (population or people or person*)) or TITLE-ABS("dependent population")) |
| ProQuest | (ti("death related") OR ti("related to death") OR ti("end of life") OR ti("terminal year*") OR ti(last PRE/4 life") OR ti("end stage of life") OR ti(final PRE/4 life") OR ti("mortality related") OR ti("related to mortality") OR ab("death related") OR ab("related to death") OR ab("end of life") OR ab("terminal year*") OR ab(last PRE/4 life") OR ab("end stage of life") OR ab(final PRE/4 life") OR ab("mortality related") OR ab("related to mortality")) AND (ab(cost*) OR ab(expenditure*) OR ab(spending*) OR ab(expense*) OR ab(financing) OR ab("financial resources") OR ti(cost*) OR ti(expenditure*) OR ti(spending*) OR ti(expense*) OR ti(financing) OR ti("financial resources")) AND (ti(aged) OR ti(elder*) OR ti(older*) OR ti(senior*) OR ti(geriatric*) OR ti(pensioner*) OR ti(65) OR ti(70) OR ti(75) OR ti(80) OR ti(85) OR ti(90) OR ti("old age*") OR ti(old PRE/1 (population OR person* OR people OR patient*)) OR ti(retired PRE/1 (population OR person* OR people)) OR ti("dependent population") OR ab(aged) OR ab(elder*) OR ab(older*) OR ab(senior*) OR ab(geriatric*) OR ab(pensioner*) OR ab(65) OR ab(70) OR ab(75) OR ab(80) OR ab(85) OR ab(90) OR ab("old age*") OR ab(old PRE/1 (population OR person* OR people OR patient*)) OR ab(retired PRE/1 (population OR person* OR people)) OR ti("dependent population")) |
